# Advancing IBDV diagnostics: a one-step multiplex real-time qRT-PCR for discriminating between vvIBDV and non-vvIBDV viruses, including the newly emerged IBDV variant

**DOI:** 10.3389/fvets.2024.1421153

**Published:** 2024-07-18

**Authors:** Amany Adel, Ali Zanaty, Zienab Mosaad, Karim Selim, Naglaa M. Hagag, Mona Badr, Hany Ellakany, Momtaz Shahien, Ahmed Samy

**Affiliations:** ^1^Reference Laboratory for Veterinary Quality Control on Poultry Production, Animal Health Research Institute, Agriculture Research Center (ARC), Dokki, Giza, Egypt; ^2^Poultry and Fish Diseases Department faculty of Veterinary Medicine, Damanhour University, Damanhur, Egypt; ^3^Infectious bursal disease virus group, the Pirbright Institute, Surrey, United Kingdom

**Keywords:** VP2, VP1, cIBDV, vvIBDV, nVarIBDV, real-time PCR

## Abstract

The very virulent infectious bursal disease virus (vvIBDV) induces an acute, highly contagious and immunosuppressive disease in younger chicken causing massive economic losses globally. A major challenge in the field’s clinical diagnosis is distinguishing gross lesions caused by vvIBDV from those induced by classic IBDV (cIBDV), commonly used as live attenuated vaccines. This study introduces a one-step multiplex real-time PCR assay designed to distinguish between vvIBDV and non-vvIBDV viruses. Via simultaneously targeting the VP2 sequence for vvIBDV detection and the VP1 sequence for non-vvIBDV identification, including classic, American variant and the recently emerged novel variant IBDV (nvarIBDV), the assay’s specificity was validated against common avian viral diseases and nonspecific IBDV strains without any observed cross-reactions. It effectively differentiated between vvIBDV and non-vvIBDV field samples, including nvarIBDV, as confirmed by genotyping based on VP2 sequencing. The assay demonstrated a limit of detection ranging from 1.9×10^10^ to 10^3^ DNA copies for vvIBDV-VP2, 9.2×10^10^ to 10^3^ DNA copies for classic strains, and 1.2×10^11^ to 10^4^ DNA copies for nvarIBDV in VP1 detection of non-vvIBDV. In conclusion, this study presents a specific, sensitive, and straight forward multiplex real-time PCR assay.

## 1 Introduction

Infectious bursal disease (IBD) is an acute, highly contagious, immunosuppressive virus affecting young chickens. This disease is characterized by the extensive destruction of immature B lymphocytes and severe damage to the cloacal bursa. Typically, high mortality rates are observed in chickens at 3 weeks of age and older for certain strains. The immune suppression caused by IBD leads to diminished responsiveness to commonly used vaccines and increases the impact of other diseases (1). It is caused by the IBD virus (IBDV), a non-enveloped, double-stranded RNA (dsRNA) virus that belongs to the genus Avibirnavirus under the family Birnaviridae and is composed of two segments (A and B) (2). Segment A (3.2 kb) is formed from two overlapped open reading frames: the first encodes VP5, while the second encodes the VP2, VP3, and VP4 polyprotein, among which VP2 and VP3 are structural proteins. VP2 has a hypervariable region (HVR) that is responsible for virus antigenicity and virulence. Segment B (2.8 Kb) encodes a viral RNA polymerase (VP1) (3, 4).

IBDV is classified into two serotypes based on antigenicity, with only serotype 1 being pathogenic in chickens (5). Serotype 1 viruses are further categorized based on pathogenicity into classical IBDV (cIBDV), variant IBDV (varIBDV), very virulent IBDV (vvIBDV), attenuated IBDV, and novel variant IBDV (nVarIBDV) (6). The virus’s bi-segmented double-stranded RNA genome and the presence of viral RNA-dependent RNA polymerase (RdRP) contribute to continuous evolution through mutations, reassortment, and recombination (7). This evolution challenges classification based on pathogenicity and hinders accurate and rapid diagnosis. Therefore, improved genotyping schemes, mainly based on VP2-HVR region sequences along with partial sequences of VP1, have been proposed to describe the genetic and pathogenic diversity of IBDV (7–10). While accurate and rapid diagnosis remains challenging, especially in differentiating between vvIBDV and classic virulent strains, including vaccinal strains, due to their similarity under field conditions. Both genotypes are associated with characteristic postmortem lesions, such as muscular hemorrhage, inflammatory exudation, hemorrhage, and yellow staining of the bursae, while mortality rates vary based on the pre-existing immunity and strains (4, 11) making differentiation challenging, especially considering the widespread use of live classical strains-based vaccines for clinical protection against vvIBDV (12).

Rapid and accurate differentiation between vvIBDV and non-vvIBDV strains is crucial to understanding the epidemiology of viral spread and the efficiency of used control strategies. Here, we propose using Real-time RT-PCR, targeting the phylogenetically relevant genes of the IBDV genome, VP1 and VP2 genes, for molecular diagnosis of IBDV infection. These approaches will enable faster, more sensitive, and specific detection and discrimination of IBDV strains compared to the currently used approach relying on RT-PCR and gene sequencing.

## 2 Materials and methods

### 2.1 IBDV variant analysis and primers and probs design

**Figure 1:**
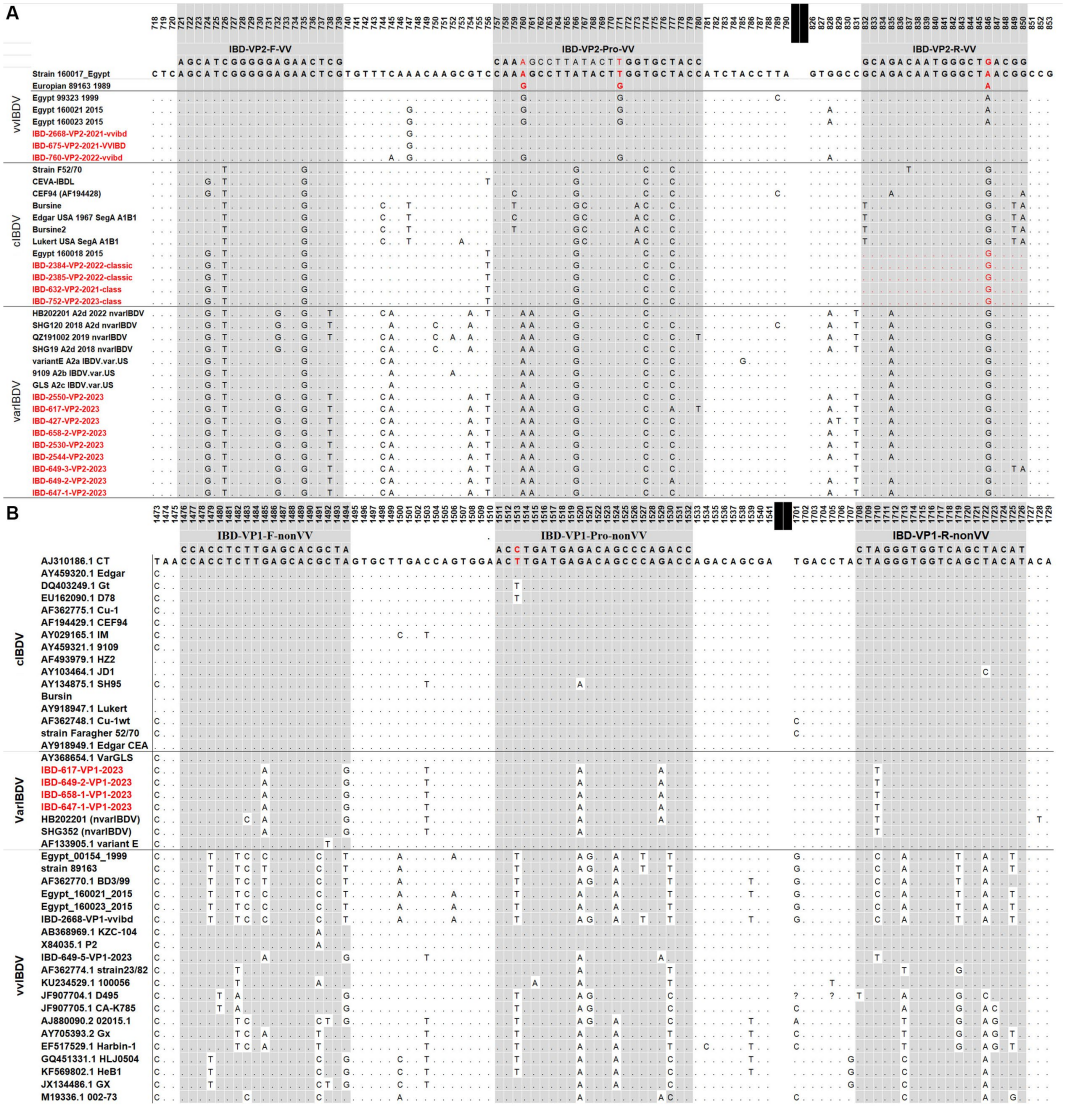
Alignment of forward, reverse, and probe sequences of the real-time RT-PCR. Various representative vvIBDV, classical (cIBDV), and variant (nVarIBDV) strains’ VP2 sequences **(A)** and VP1 sequences **(B)**. The primers and probes target sites are indicated using the European vvIBDV strain 89163 for VP2 and the classical strain CT for VP1.

The primers and probes were designed *in silico* using the nucleotide sequences of IBDV’s VP1 and VP2 genes. In brief, several aligned nucleotide sequences for each of the VP1 and VP2 genes from the classic, variation, variant, and vvIBDV strains of Egyptian viruses under research, as well as additional reference strains retrieved from the NCBI database, were employed. The sequences were aligned with Bioedit software (13) and examined to identify the most specific locations for vvIBDV on the VP2 gene and classic strains on the VP1 gene (Figure 1). The specificity, secondary structures, and other physical properties of the proposed primers and probes were analyzed *in silico* using BLAST on the NCBI and OligoCalc servers.

### 2.2 Field samples and vaccinal strains used in validation of the assay

Bursa samples from forty-six vaccinated broiler farms that were submitted to our laboratory to confirm clinical diagnoses have been used to evaluate the efficiency of the assay. Sample preparation, following the method outlined in (14), Briefly, homogenizing the bursae with phosphate-buffered saline supplemented with Kanamycin (700 μg/mL). The homogenate was then subjected to three cycles of freezing and thawing, followed by centrifugation at 8,000 rpm for five minutes to remove cell debris. The resulting supernatant was collected, filtered through a 0.45 μm filter (Sartorius, Germany), and stored in a −80°C freezer for the next step. Also, Leukert, Winterfeild, JOVAC (D78) and Bursin vaccinal strains were used in validation of the assay.

### 2.3 RNA extraction and one step RT-qPCR

Viral RNA extraction was conducted using the EasyPure® Viral DNA/RNA Kit (TransGen Biotechnology, Inc., Beijing, China), following the kit instructions. Subsequently, cDNA synthesis and qPCR were performed using the TransScript® Probe One-Step qRT-PCR SuperMix (TransGen Biotechnology, Inc., Beijing, China), following the manufacturer’s instructions. The one-step real-time PCR was performed and analyzed in a Stratagene Mx3000P instrument (Agilent Technologies Inc., Santa Clara, CA, USA) using primers and probes targeting VP2-HVR and VP1 (Table 1). The thermal profile was as follows: Reverse transcription at 45°C for 15 min, initial denaturation at 94°C for 5 min, followed by 40 cycles of 94°C for 40 s and 52°C for 60 s.

**Table 1:** primers and probes of the multiplex real time RT-PCR assay and the primers of nucleotide sequencing assay.

Oligonucleotide ID	Sequence (5′ – 3′)	Site and/or product size	Assay
IBD-VP2-F-VV	AGCATCGGGGGAGAACTCG	721–739	vvIBDV specific Real Time PCR
IBD-VP2-R-VV	CCGTCAGCCCATTGTCTGC	832–850	
IBD-VP2-Pro-VV	HEX-CAAAGCCTTATACTTGGTGCTACC	757–780	
IBD-VP1-F-nonVV	CCACCTCTTGAGCACGCTA	1,590–1,608	non-vvIBD specific Real Time PCR
IBD-VP1-R-nonVV	ATGTAGCTGACCACCCTAG	1,822–1,840	
IBD-VP1-Pro-nonVV	FAM-ACCTGATGAGACAGCCCAGACC	1,625–1,646	
VP2-F1	GGATACGATCGGTCTGACCCC	1,910 bp (vvIBDV)	Sensitivity assay
VP2-R1910	CGTATGAAGGATCCTCTTTGAGA		
VP1-F1345	ACACGTGGTACTCAATTGACCTA	650 bp (non vvIBDV)	
VP1- R2000	TACCAACCTCAACGCCTCATACC		
VP2-F673	GTAACAATCACACTGTTCTCAGC	672 bp	VP2 gene sequencing
VP2-R1345	TTATGTCTTAGAAGCCAAATGC		
VP2-F445	GCCAACATCAACGACAAAAT	450 bp	
VP2-R898	ATTGGCTGGGTTATCTC		
VP1-F320	GAGAATGAGGAGTATGAGACCGA	750 bp	VP1 gene sequencing
VP1-R1150	GAGATCATGAGGTGTGTTGG		
VP1-F750	GGGCTTGTCATCCTCACCGG	400 bp	
VP1-R1750	GAGATCATGAGGTGTGTTGG		

### 2.4 Specificity and sensitivity of the primers and probes

The specificity of the designed primers and probes for the multiplex assay was tested using RNA from known IBDV strains representing different genotypes, as well as other common viral pathogens in the poultry population such as LPAI-H9N2, HPAI-H5N8, IBV, and NDV, employing one-step real-time PCR. To assess the sensitivity of the assay, specific purified PCR products were amplified using specific primers (Table 1) and the Easyscript® One-Step RT-PCR kit (TransGen Biotech) following the manufacturer’s recommendations. The copy numbers in the purified products were calculated as described in,^1^ followed by tenfold serial dilution of one or multiple samples, which were then subjected to single or multiplex real-time PCR assays. The purified amplified PCR products of VP2 from the sample IBD-2668-VP2-2021-vvIBDV and VP1 from IBD-632-VP1-2021-classic and IBD-2530-VP1-2023-new variant were utilized to determine the sensitivity limit of the assay.

### 2.5 Field sample PCR testing and genotyping confirmation

The developed multiplex one-step real-time PCR was employed to test 46 clinical samples, and results were recorded. To validate the results, representative samples of each genotype underwent partial gene sequencing of the VP1 and VP2-HVR genes. For the amplification, specific primers (Table 1) and the Easyscript® One-Step RT-PCR kit (TransGen Biotech) were used following the manufacturer’s recommendations. Subsequently, the PCR products at the expected size were purified using the QIAquick Gel Extraction Kit (QIAGEN, USA) in accordance with the manufacturer’s instructions. The BigDye Terminator kit (Applied Biosystems, CA, USA) was utilized for Sanger sequencing of the purified amplified products of both VP1 and VP2 genes. The prepared sequence reactions were then read by ABI (Applied Biosystems 3500xl Genetic Analyzers, USA).

### 2.6 Statistical analysis of the data

Concordance between the results of both systems and the results of the nucleotide sequences has been determined by Cohen’s Kappa agreement measure at a *p*-value <0.05. Additionally, the coefficient of determination (R^2^) in linear regression was calculated for the standard curves of the real-time qRT-PCR systems. Statistical analysis has been done by SPSS software (IBM).

## 3 Results

### 3.1 *In silico* validation and specificity of the multiplex real-time qRT-PCR

**Figure 2:**
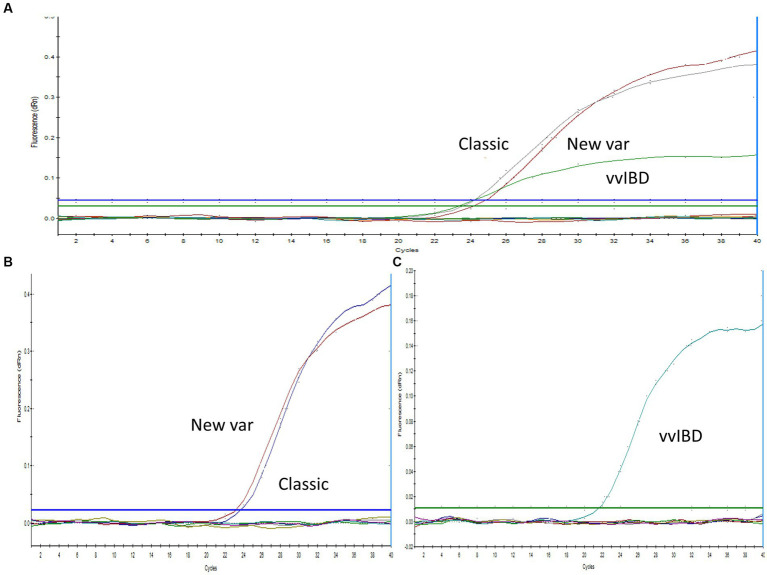
Specificity of the one-step real-time TaqMan RT-PCR assay. **(A)** Multiplex PCR analysis with positive samples representing vvIBDV, classical, and variant strains (as indicated in the figure), in addition to IBV, NDV, H9N2, and a no-template control. IBDV-negative field samples exhibited no amplification, with vvIBDV specifically recognized by the HEX fluorescence channel, while classical and variant IBDV were identified solely by the FAM fluorescence. **(B)** Uniplex PCR with positive samples representing classical and variant IBDV strains (as indicated in the figure), along with IBV, NDV, H9N2, and a no-template control. IBDV-negative field samples showed no amplification, with classical and variant IBDV uniquely identified by FAM fluorescence. **(C)** Uniplex PCR with positive samples representing vvIBDV strains, along with IBV, NDV, H9N2, and a no-template control. IBDV-negative field samples did not exhibit amplification, and vvIBDV was specifically recognized by HEX fluorescence.

Sequence analysis was conducted on the VP2-HVR sequences of several IBDV strains to identify nucleotide polymorphisms that could distinguish between vvIBDV and non-vvIBDV sequences. Sequences spanning from nucleotide 721 to 850 has some unique nucleotide sequences to vvIBDV strains and can be employed for differentiation (Figure 1A). The selected probe, along with the forward and reverse primer sequences, exhibited 3 to 6, 3 to 5, and 2 to 4 single nucleotide polymorphisms (SNPs), respectively, in selected representative non-vvIBDV strains when compared to vvIBDV, including the Egyptian antigenically atypical vvIBDV (Figure 1A). On the other hand, sequences spanning from nucleotide 1,476 to 1,726 in segment B can be used to distinguish between classic and variant strains compared to vvIBDV, with 2 to 6 single nucleotide polymorphisms (SNPs) in vvIBDV compared to classical and variant strains (Figure 1B). It’s worth mentioning that when aligning the designed primers and probes used to distinguish non-VVIBDV with nVarIBDV sequences, there are 1–2 SNPs in the primer and probe sequences (Figure 1B). Since the system was primarily established to differentiate between vvIBDV and non-vvIBDV, which can manifest with similar clinical symptoms and lesions, nVarIBDV, known to induce subclinical infection, can be easily distinguished from vvIBDV. Technically, the developed multiplex qrt-PCR assay showed no signal with viruses and live attenuated vaccines commonly found on poultry farms, including HPAI-H5, LPAI-H9N2, IBV, and NDV, and the negative or no template controls in the assay were also not amplified. Additionally, the uniplex assay that was specific for vvIBDV showed negative results for caIBDV and nVarIBDV, and vice versa (Figure 2). Since the antigenically atypical vvIBDV has been only recognized by HEX fluorescence channel, while the classic and the nvarIBDV have been recognized only by FAM fluorescence channel, indicating no nonspecific binding with non-matching primer and probe combinations (Figure 2).

### 3.2 Sensitivity and limit of detection

**Figure 3:**
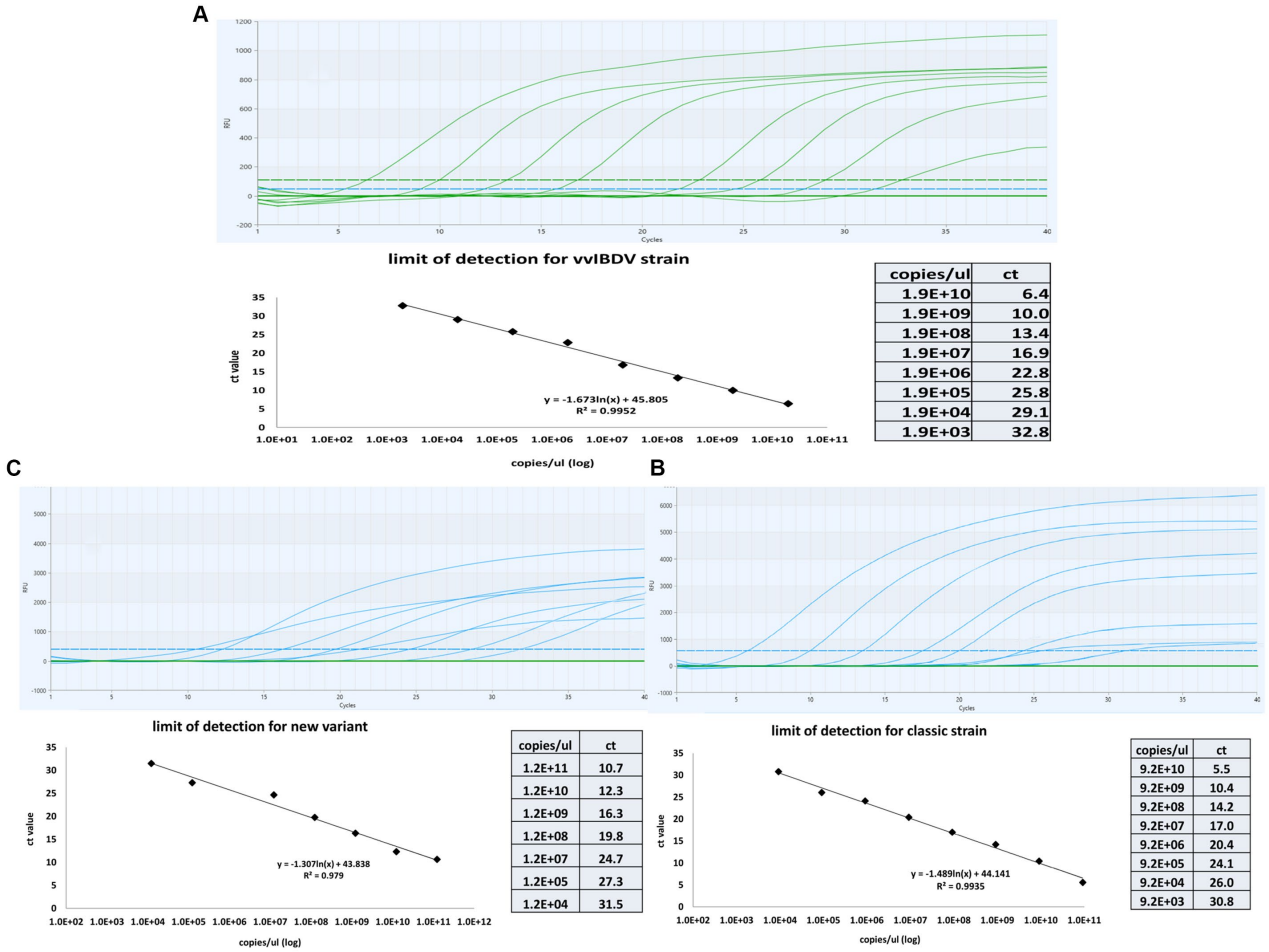
Sensitivity and standard curves for the multiplex one-step real-time-TaqMan RT-PCR assay. The curves were constructed by employinga 10-fold serial dilution of known concentrations of genetic material from samples representing very virulent **(A)**, classical **(B)**, and new variant IBDV **(C)**. The figure demonstrates the linear correlation between Cycle Threshold (CT) and the quantity of genetic material for different IBDV genotypes. The coefficient of determination (R_2_) and efficiency of each linear regression curve are indicated.

Tenfold serial dilutions of known concentrations of genomic material for each genotype were tested using the multiplex one-step real time-PCR to establish a correlation between the cycle threshold (CT) and the quantity of IBDV genetic material. The results revealed linear correlation between CT and IBDV genetic quantity with an average R2 of 0.99 for vvIBDV and caIBDV strains and 0.979 for nVarIBDV (Figure 3). The detection limit for vvIBDV was in the range of 1.9×10^10^ to 1.9×10^3^ DNA copies/μL. Regarding the non-vvIBDV, the limit of detection ranged from 9.2×10^10^ to 9.2×10^3^ DNA copies/μL for the classic strain, while for the nVarIBDV, the range was 1.2×10^11^ to 1.2×10^4^ DNA copies/μL (Figure 3). Each dilution of each strain was tested in triplicate, and then the averages of these reads have been calculated and used as points for evaluation of the limit of detection for each strain, as illustrated in (Figure 3).

### 3.3 Molecular detection of field samples by multiplex RT-qPCR assay

Forty-six bursa samples, along with vaccinal strains commonly used for vaccination in Egyptian poultry farms, including Lukert, Winterfield, Bursin, and D78 strains, have been examined by a multiplex one-step real-time TaqMan RT-PCR assay developed in the present manuscript (Table 2). Samples were collected from farms with ages ranging from 20 to 34 days, exhibiting varied clinical lesions—from severe hemorrhage in the bursa, thigh, and breast muscles accompanied by typical IBDV clinical signs to lesions limited to atrophy in the bursa with no apparent clinical symptoms. Based on the multiplex one-step real-time TaqMan RT-PCR results, 8 samples were positive for vvIBDV, 37 were positive for non-vvIBDV, and 5 samples were positive for both vvIBDV and non-vvIBDV (Table 2).

**Table 2:** Clinical samples analyzed using multiplex one-step real-time TaqMan RT-PCR.

No.	Sample ID	Age (days)	date of collection	Vaccination	The multiplex one-step RT-PCR		Genotype based on VP2-HVR	VP2 accession No
					Non vvIBD (CT)	vvIBD (CT)		
1	2668	23	11/06/2021	Transmune ibd	Negative	22.62	vvIBDV	OR506930
2	760	27	24/05/2023	VAXXITEK	Negative	22.09		OR506938
3	675	28	15/02/2021	Transmune ibd	Negative	22		OR506931
4	2384	26	24/05/2022	Transmune ibd	20.04	Negative	Classic IBDV	OR506935
5	2385	26	24/05/2022	Transmune ibd	22.34	Negative		OR506937
6	632	24	12/02/2021	Transmune ibd	16	Negative		OR506939
7	752	21	24/03/2023	VAXXITEK	21.08	Negative		OR506940
8	Lukert	N/A	NA	NA	25.26	Negative		AY918948
9	Winterfield	N/A	NA	NA	25.61	Negative		MH329181
10	Bursin	N/A	NA	NA	30.72	Negative		KY610532
11	D78	N/A	NA	NA	36.09	Negative		EU162087
12	617	24	01/06/2023	VAXXITEK	22.21	Negative	nVar-IBDV	OR506921
13	647-1	20	01/06/2023	VAXXITEK	18.07	Negative		OR506920
14	649-2	21	01/06/2023	VAXXITEK	22.91	Negative		OR506922
15	658-2	28	04/06/2023	VAXXITEK	18.77	Negative		OR506923
16	2530	21	05/06/2023	VAXXITEK	22.05	Negative		OR611974
17	2544	23	05/06/2023	VAXXITEK	26.22	Negative		OR611975
18	2550	35	08/06/2023	VAXXITEK	28.23	Negative		OR611976
19	649-3	22	01/06/2023	VAXXITEK	28.71	Negative		OR506924
20	427	23	01/06/2023	VAXXITEK	18.46	Negative		OR506925
21	518	23	03/02/2021	Transmune ibd	20.07	20.23	ND	ND
22	522	28	03/02/2021	Transmune ibd	22.12	28.56		ND
23	526	22	03/02/2021	Transmune ibd	24.09	26		ND
24	671	28	15/02/2021	Transmune ibd	22.04	22.63		ND
25	677	28	15/02/2021	Transmune ibd	20.67	20.01		ND
26	751	21	24/05/2023	VAXXITEK	Negative	20.38		ND
27	756	28	24/04/2023	VAXXITEK	26.11	Negative		ND
28	757	28	24/04/2023	VAXXITEK	28.59	Negative		ND
29	758	28	24/04/2023	VAXXITEK	28.93	Negative		ND
30	759	28	24/04/2023	VAXXITEK	19.03	Negative		ND
31	761	27	24/05/2023	VAXXITEK	Negative	18.02		ND
32	2526	21	05/06/2023	VAXXITEK	24.43	Negative		ND
33	2531	28	05/06/2023	VAXXITEK	26.39	Negative		ND
34	2556	34	08/06/2023	VAXXITEK	28.09	Negative		ND
35	2576	27	10/06/2023	VAXXITEK	28.41	Negative		ND
36	2580	27	10/06/2023	VAXXITEK	28.12	Negative		ND
37	2603	35	11/06/2023	VAXXITEK	28.05	Negative		ND
38	2613	20	14/06/2023	VAXXITEK	26.02	Negative		ND
39	2634	18	14/06/2023	VAXXITEK	30.12	Negative		ND
40	2691	27	17/06/2023	VAXXITEK	28.12	Negative		ND
41	2683	22	NA	VAXXITEK	26.34	Negative		ND
42	2697	22	NA	VAXXITEK	24.05	Negative		ND
43	753	21	24/03/2023	VAXXITEK	Negative	20.01		ND
44	754	28	24/04/2023	VAXXITEK	Negative	26.32		ND
45	755	28	24/04/2023	VAXXITEK	Negative	16.11		ND
46	649-5	20	13/06/2023	VAXXITEK	20.9	Negative		ND
47	658-1	28	13/06/2023	VAXXITEK	21.05	Negative		ND
48	658-3	28	04/06/2023	VAXXITEK	16.29	Negative		ND
49	649-4	22	01/06/2023	VAXXITEK	23.34	Negative		ND
50	649-6	21	13/06/2023	VAXXITEK	18.26	Negative		OR506936

NA, not available; ND, not detected.

### 3.4 Nucleotide sequence and phylogenetic analysis

**Figure 4:**
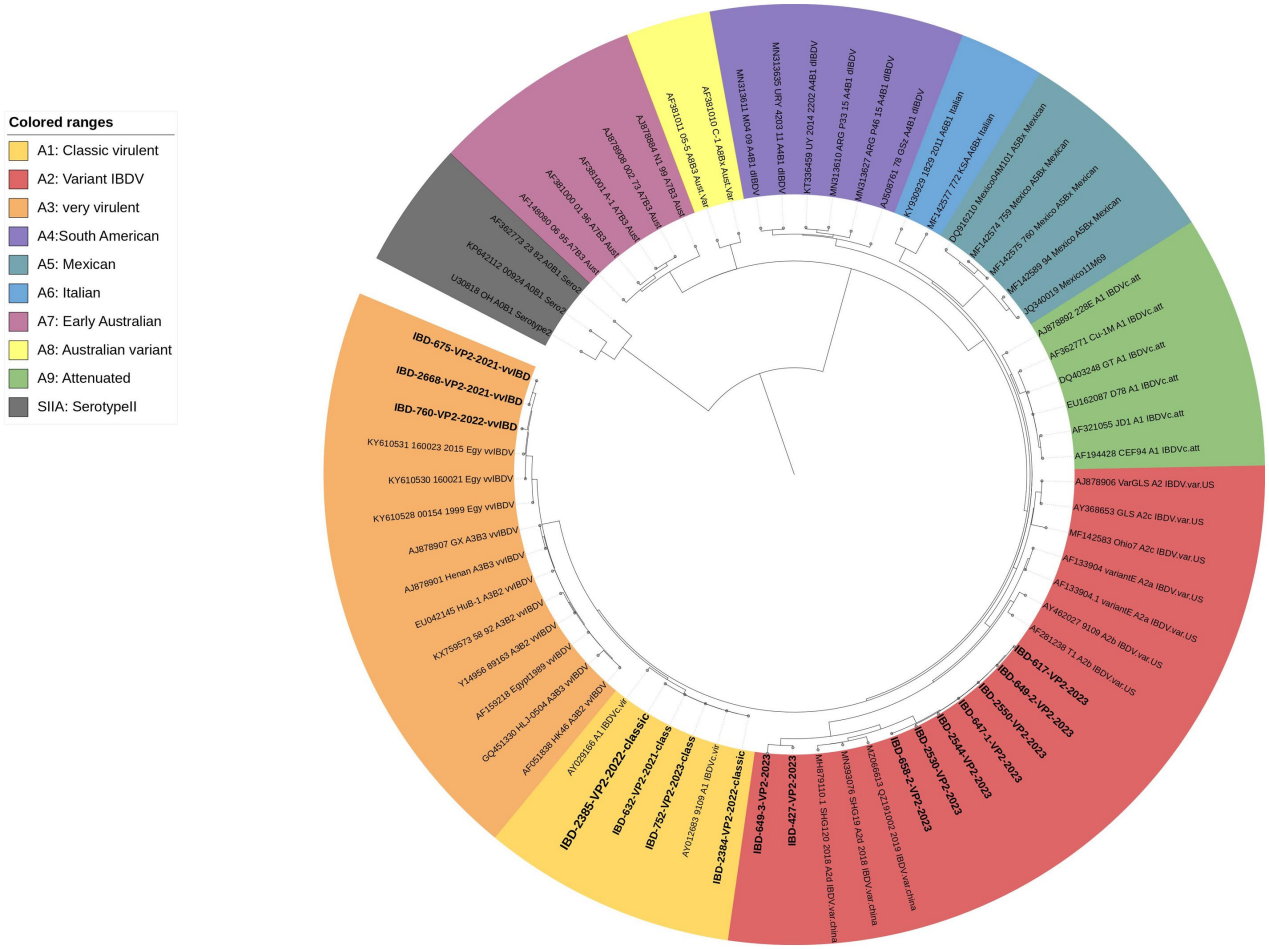
A concise circular phylogenetic tree based on the VP2-HVR sequence. The tree was generated using a maximum likelihood (ML) with 1,000 bootstrap replications, using the IQ-TREE program (17) and the (GTR + F + G4) substitution model embedded within IQ-Tree. The tree was annotated in iTOL (https://itol.embl.de) with genogroup information displayed in colored circular using scheme proposed by Gao et al. (7). Samples tested in the present study are in bold.

The results of the phylogenetic analysis based on VP2-HVR revealed that, out of the 17 successfully sequenced samples, 3 clustered within the A3 genogroup representing vvIBDV, 4 clustered within the A1 genogroup representing classic virulent IBDV, and 9 clustered within the A2 genogroup representing variant IBDV and subgroup d, which represents the newly emerged variant strains (nvarIBDV) (Figure 4).

**Figure 5:**
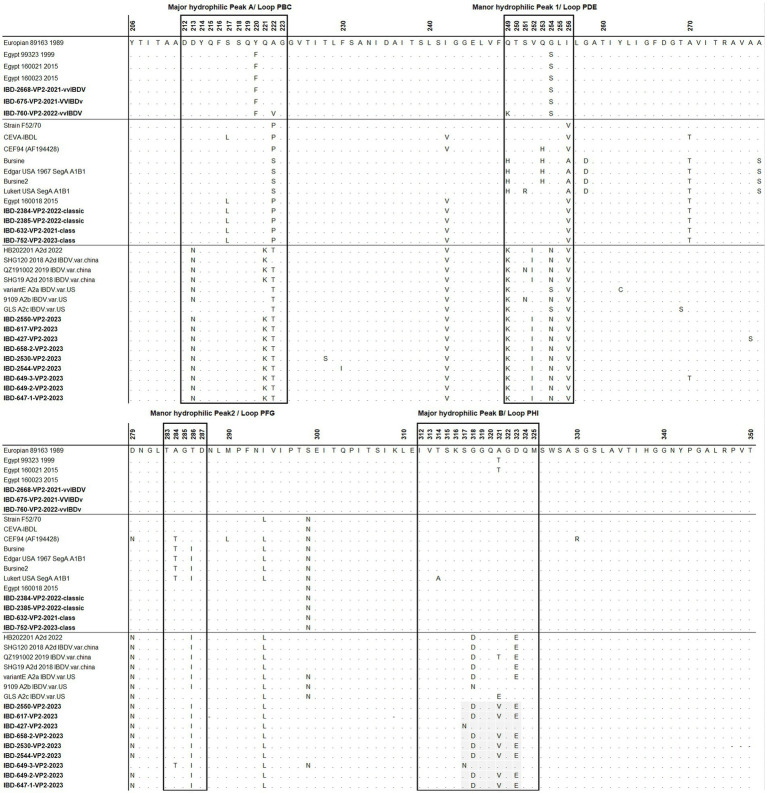
Amino acid alignment of VP2 HVR for the successfully sequenced samples in comparison with reference sequences. Amino acids surrounded by black line boxes indicate the most exposed peaks of the VP2 P domain and amino acids surrounded by red dotted lines red boxes represent unique amino acid mutation for the newly emerged nVarIBDV in Egypt. Samples tested in the present study are in bold.

The deduced amino acid sequences of VP2 for the studied samples were aligned with reference samples representing each genogroup (Figure 5). All Egyptian variant samples exhibited typical residues found in variant IBDV, including 222T, 249K, 286I, and 318D, along with the three conserved amino acid residues found only in Chinese variant IBDVs: 217K, 252I, and 299S. However, two samples, namely 427 and 649, possessed 318G and 323D, resembling the GLS strain at these residues. In addition, sample 649-3 exhibited 299N, resembling GLS a9ariantntE strains. Apart from samples 427 and 649, all Egyptian variant strains possessed 321V (Figure 5).

Regarding classical strains, the amino acid sequences for VP2-HVR of cIBDV showed typical amino acid similarity with IBDL, a commonly used vaccine strain in Egypt (Figure 5). In comparison to the Egyptian antigenic atypical vvIBDV strain isolated in 1999, all the vvIBDV samples detected in the present study possessed Y220F and G254S located in major hydrophilic peak A and manor hydrophilic peak 1, respectively, but not A321T in major hydrophilic peak B (Figure 5).

### 3.5 Agreement between the in-house multiplex real-time qRT-PCR and genotyping by nucleotide sequence

Only 17 samples were successfully sequenced to cover the entire VP2-HVR, which is required for genotyping (Figure 4; Table 2). Comparing the results of genotyping with the established one-step real-time PCR revealed 100% concordance with genotyping based on gene sequencing, and it also agreed with the clinical findings. It is worth mentioning here that the established one-step real-time PCR was able to detect the nVarIBDV samples newly emerging in Egypt with no marked CT value difference between nVarIBDV and cIBDV.

## 4 Discussion

Infectious bursal disease represents a significant threat to poultry production globally, characterized by mortality rates that can reach up to 100% and immunosuppression in young chicks (15). With the emergence of vvIBDV and classical strain-based live IBDV vaccines, they have become widely utilized in numerous countries, including Egypt (12, 16). Some of these strains target the bursa and cause lesions that add more complications to field diagnosis, especially if we take into account the common lesions induced by classic IBDV and vvIBDV (4, 11). The differentiation between various IBDV field strains and vaccinal strains relies mainly on gene sequencing of the VP2-HVR region. However, gene sequencing is a laborious, time-consuming, and expensive process, further complicated by supply chain issues, especially in low- and middle-income countries. Here, we designed and validated a one-step real-time qRT-PCR using two distinct and specific probes, along with two sets of primers simultaneously, to distinguish between vvIBDV and non-vvIBDV strains. One set of primers and a HEX-labeled TaqMan probe are specific to vvIBDV VP2-HVR, while the non-vvIBDV strains are identified by a FAM-labeled TaqMan probe and two primers specific to the VP1 gene of the classic and variant IBDV strains. The proposed real-time qRT-PCR assay not only saves time and is affordable but also can detect mixed infections with more than one genotype.

Numerous trials and assays have been conducted for the rapid detection and identification of classic and very virulent strains of IBDV. These include nanoparticle-assisted PCR, SYBR green, and TaqMan-based real-time RT-PCRs (RT-qPCR) (18–21). Including a recent study developed a real-time one-step qRT-PCR assay, this assay can distinguish the nVarIBDV from other non-nVarIBDV strains based on nucleotide polymorphisms in the 5’-UTR and the vp5/vp2 overlapping region of the segment A sequences (22). Despite the widespread use of the nVarIBDV in several countries, including China, Japan, South Korea, Malaysia, and Egypt (6, 23–26) the vvIBDV remains a significant threat to poultry production (25, 27) due to its high replication efficiency and rapid spread (4) and its rapid accurate differentiation from classical strains ultimately required to assess the vaccination efficiency. Previous studies, such as those by Gao et al. (7) and Michel and Jackwood (9), have also focused on distinguishing IBDV strains. However, in our assay in addition to that our assay successfully differentiates newly emerged variant strains, providing a rapid and accurate single-assay solution for field diagnostics.

Results showed that the designed assay had no cross-reaction with other circulating viruses in the field, such as HPAI H5N8, LPAI H9N2, IBV, and NDV. Using a tenfold serial dilution of known amounts of VP1 and VP2 sequences, the assay detected up to 1.9×10^3^, 9.2×10^3^, and 1.2×10^4^ DNA copies/μL at CT 32.8, 30.78, and 31.45 for vvIBDV, classic IBDV, and nvarIBDV, respectively. The assay successfully differentiated between vvIBDV and non-vvIBDV field samples with a zero-error rate compared to sanger sequencing results, regardless of the mismatches, especially in the probe in different vvIBDV strains.’ It’s essential to note that, due to the high variability of VP2-HVR, some mismatches occurred with different vvIBDV strains, including some Egyptian antigenically atypical and typical vvIBDV. Yet, there was no marked impact on the assay sensitivity, as indicated by the CT values of the samples tested. Despite potential mismatches in viral field isolates that could impact quantification, leading to false negatives, the impact of mismatches on PCR efficiency varies based on sequence context and reaction conditions. For example, mismatches toward the end of the primers can dramatically impact the PCR efficiency, but in some cases, they can also contribute to the amplification efficiency and may avoid false priming (28).

While our work is ongoing, a new Chinese variant (nVarIBDV) emerged in Egypt in early 2023. This variant, first recorded in China in 2019, belongs to genotype A2dB1 (6, 7). Despite mismatches, especially in forward primers and probes, the FAM-labeled assay successfully detected the Chinese nVarIBDV. In general, the assay was able to detect all field samples with a relatively low CT ranging from 18–28, indicating its suitability for field situations.

VP2-HVR sequencing is considered phylogenetically representative of the full genome length of IBDV and is used for genotypic classification (7–10). Here in the present study we used genotyping scheme proposed by Gao et al. (7) to confirm the results of real time PCR. Phylogenetic analysis revealed three samples clustered with vvIBDV and other Egyptian antigenically atypical vvIBDV that originally emerged in 1999. Four samples clustered with classic virulent strains, showing high similarity with hot strain (IBDL) live vaccines. Nine samples clustered with the nvarIBDV genogroup that emerged in Egypt in 2023, showing high similarity with Chinese variant strains (25). It’s worth mentioning that only 17 samples out of 46 were successfully sequenced from field material, highlighting the challenge of genotyping IBDV based on Sanger sequencing. However, our in-house, one-step real-time qRT-PCR was found to be capable of detecting single and mixed infections of various genotypes directly from field samples.

## 5 Conclusion

The persistent threat posed by vvIBDV to poultry production necessitates the development of a rapid and sensitive assay to distinguish between vvIBDV and non-vvIBDV strains that are commonly used as vaccination strains. Here, we propose a real-time qRT-PCR assay using two sets of primers and probes simultaneously. The first set detects vvIBDV based on VP2-HVR, and the second set detects non-vvIBDV genotypes based on VP1 sequencing of classic and American variant IBDV. Despite the high variability of HVR, the designed primers and probes showed higher sensitivity with the circulated vvIBDV in Egypt. We also report the emergence of nvarIBDV that can be recognized by non-vvIBDV primers and probes. It is necessary to highlight here that the assay has been thoroughly tested using vvIBDV samples collected from Egypt, considered an antigenic atypical vvIBDV. More samples representing different vvIBDV strains are required to be tested.

## Data availability statement

The Nucleotide sequence of the tested field samples presented in this study have been deposited in GenBank. The accession numbers are provided in the article.

## Ethics statement

The animal study was approved by the Scientific and Ethics Committee of the Animal Health Research Institute (AHRI), Agriculture Research Center (ARC), Egypt. The study was conducted in accordance with the local legislation and institutional requirements.

## Author contributions

AA: Conceptualization, Data Curation, Investigation, Methodology, Validation, Writing – original draft, Writing – review & editing. AZ: Formal analysis, Methodology, Writing – original draft. ZM: Formal analysis, Software, Validation, Writing – original draft, Writing – review & editing. KS: Investigation, Writing – original draft. NH: Investigation, Writing – review & editing. MB: Methodology, Writing – original draft, Writing – review & editing. HE: Writing – review & editing. MS: Writing – review & editing. AS: Formal analysis, Data Curation, Investigation, Writing – original draft, Writing – review & editing.

## Footnotes

^1^ https://www.technologynetworks.com/tn/tools/copynumbercalculator
